# A cross-sectional study of *Staphylococcus aureus* reservoirs in dairy goats, their environment and farm workers

**DOI:** 10.1016/j.onehlt.2026.101624

**Published:** 2026-10-17

**Authors:** Mariam F.M. Adetunji, Maxime A.M. Pals, Kacper Libera, Andries A. Kampfraath, Gerrit Koop, Manouk Vrieling, Lidwien A.M. Smit

**Affiliations:** a Sustainable Ruminant Health, Department of Population Health Sciences, Faculty of Veterinary Medicine, Utrecht University, Yalelaan 7, 3584 CL, Utrecht, the Netherlands; b Institute for Risk Assessment Sciences (IRAS), Department of Population Health Sciences, Faculty of Veterinary Medicine, Utrecht University, Yalelaan 2, 3584 CM, Utrecht, the Netherlands; c Wageningen Bioveterinary Research, Houtribweg 39, 8221 RA, Lelystad, the Netherlands

**Keywords:** Airborne dust, Mastitis, Dairy goat, Udder health, Whole genome sequencing, Sequence types, One health

## Abstract

*Staphylococcus aureus* (*S. aureus*) induced mastitis in dairy goats is a serious concern affecting animal health and welfare and imposing economic burdens on dairy goat farms. The reservoirs and possible transmission routes of *S. aureus* on dairy goat farms are not fully understood. This study examined *S. aureus* reservoirs on Dutch dairy goat farms using a One Health approach. Samples (*n* = 329) were obtained on 29 dairy goat farms from goat noses (*n* = 145), farm workers' hands (*n* = 80), and milking parlour teat cups (*n* = 54). Environmental airborne dust samples (*n* = 50) were collected using electrostatic dust collectors (EDCs). Samples were cultured and analysed with the MALDI-TOF (Matrix-Assisted Laser Desorption/Ionization, Time-Of-Flight). *Staphylococcus aureus* was detected on 27 farms, with about 27% of samples positive. Detection rates were highest in EDCs, confirming airborne dust as a major reservoir and potential transmission route. Hand swabs were positive least frequently, but indicated occupational exposure, particularly among milkers and farm owners. Glove users had a higher proportion of positive hand swabs compared to non-users. Participants who neither entered the stable nor handled goats tested negative. Whole genome sequencing identified ST133 as the predominant sequence type (76% of isolates), whereas ST398 was detected in 10% of the isolates. Isolates clustered mainly by farm and across all sample types. Overall, results indicate that *S. aureus* is present on nearly all included dairy goat farms, with positive samples in goats, on human hands, and in the environment. Preventive measures should emphasise biosecurity, hygiene, and monitoring.

## Highlights

• *Staphylococcus aureus* is an important cause of mastitis in goats.
• *Staphylococcus aureus* was found in 27 out of 29 dairy goat farms.
• *Staphylococcus aureus* was detected in goats, humans and the farm environment.
• Goat farm airborne dust samples were most often positive for *Staphylococcus aureus*.
• Whole genome sequencing identified ST133 as the predominant sequence type.

## Graphical abstract

**Figure:**
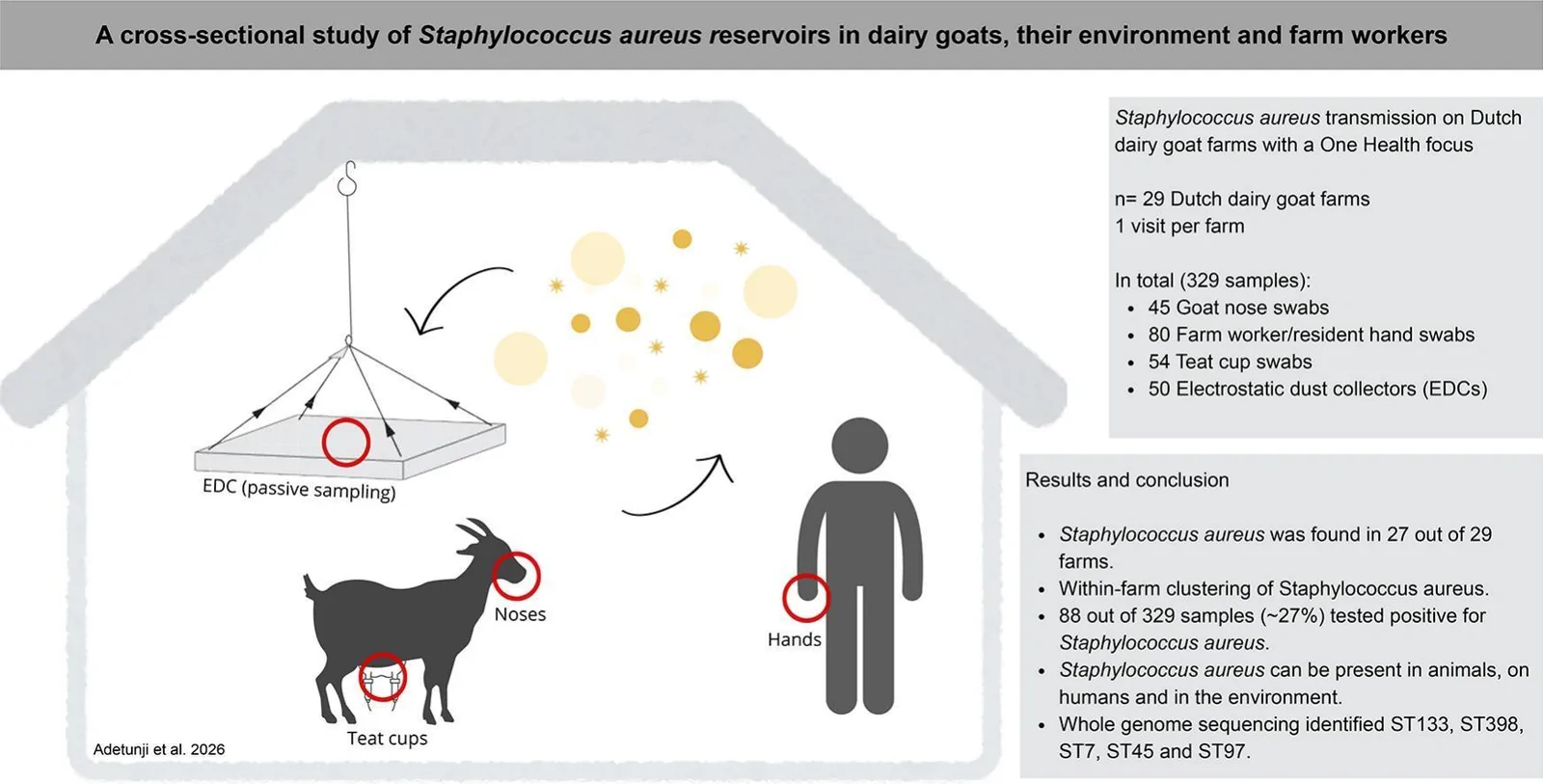

## 1 Introduction

*Staphylococcus aureus* (*S. aureus*) is a zoonotic pathogen that can harm dairy goats and humans [1], [2], [3]. In dairy goats, *S. aureus* can cause an intramammary infection, which can evolve into clinical mastitis (CM), an inflammation of the mammary gland [3], [4]. Subclinical and clinical mastitis cause high bacterial counts in the goats' milk, resulting in reduced quantity and quality of the milk [3], [4], [5]. Furthermore, mastitis negatively affects the goats' welfare and dairy goat sector economics, and in severe cases can lead to mortality among affected goats [3], [4], [6]. A milk yield loss of 0.13 kg/day was associated with *S. aureus* intramammary infections in dairy goats [7]. This bacterium can be found on the dairy goats' extramammary reservoirs: udder and teat skin, and on the mucous membranes of their vagina and especially in the nose [8], [9], [10], [11]. Pals et al. [11] found that, in 32.4% out of 71 reviewed studies, the proportion of subclinical mastitis in dairy goats caused by *S. aureus* was higher than 10% in dairy goats. A review by Tibebu et al. [6] reported that *S. aureus* accounted on average for 11% of the identified mastitis associated pathogens. The relatively high prevalence of *S. aureus* underscores the need for preventive strategies aimed at reducing the occurrence of both clinical and subclinical mastitis in dairy goat herds.

*Staphylococcus aureus* can be transmitted from goat to goat or goats to humans in various ways. Physical contact between goats is a major risk for *S. aureus* transmission [9]. Previous research found *S. aureus* in goats and dairy farm workers [8], [12], [13]. The reviews by Song et al. [14] and Solberg [15] suggest the possibility of indirect transmission from goat to goat or goat to human via contaminated objects, surfaces, and airborne dust particles. For example, humans touching dairy goats during milking could contribute to direct transmission from goat to farmer and indirect transmission from goat to goat [8], [12], [16], [17]. *Staphylococcus aureus* consists of multiple genetic lineages that can be classified into sequence types (ST) [18]. ST133 is most associated with small ruminants but has also been detected in humans [19], [20].

In humans, *S. aureus* is often found in nasal mucosa and skin [21]. Approximately 30% of individuals are permanently colonised with *S. aureus*, and most individuals who are colonised will not develop an invasive infection [22]. When someone is colonised with *S. aureus*, they are asymptomatic, but it is possible to develop an infection when the colonising organisms are pathogenic [1]. As an opportunistic pathogen, *S. aureus* can cause infection when host defences are compromised, or the skin barrier is breached [22]. *Staphylococcus aureus* can cause a variety of infections, such as skin and soft tissue infections, or even more severe health issues such as lung infections, bacteremia, osteomyelitis, endocarditis and even sepsis [23], [24]. *Staphylococcus aureus* is one of the most common causes of infections in humans and is especially dangerous because it can acquire resistance against antibiotics [25]. *Methicillin-resistant Staphylococcus aureus (MRSA)* infections in humans (which have also been reported in dairy goats [26]) contribute to increased health-care costs [27], [28]. Overall, *S. aureus* is a common human pathogen that can spread easily and cause diseases.

Previous research identified *S. aureus* in both dairy goats and humans; however, there is limited research on the reservoirs, environmental aspects and potential transmission routes of *S. aureus* on dairy goat farms. This study hypothesises that the environment within the farm can play a role in the spread of *S. aureus*. In this study, the term reservoir follows the definition used by the Centers for Disease Control and Prevention (CDC), referring to an environment or habitat in which a pathogen can live and multiply, including humans, animals, or environmental sources [29]. We aim to investigate the prevalence of *S. aureus* in different reservoirs on Dutch dairy goat farms using a One Health approach. Previous studies suggested potential transmission routes [11]; however, exact transmission pathways remain unknown. Distinguishing *S. aureus* types using whole genome sequencing (WGS) could help to better understand possible relationships between reservoirs. Eventually, this information can help to strengthen farm biosecurity and reduce economic losses in the dairy goat sector. Therefore, this research will compare animal, human, and environmental *S. aureus* isolates using the outcomes of the WGS data.

## 2 Materials and methods

### 2.1 Study design

In this cross-sectional study, farms included were those that had specifically expressed interest in udder health research on their farms by responding to a survey that was distributed among dairy goat farmers of the Nederlandse GeitenZuivel Organisatie (NGZO) in 2023. The sampling was conducted on 29 dairy goat farms across the Netherlands (see Supplementary material 1), with an average herd size of 1,127 lactating goats. All participating farm owners provided written informed consent, authorising sample collection on their farms and from their employees. After collection, the samples were pseudonymised. Sampling took place from February 2025 until May 2025. In two instances, entry to the farm was not permitted by the farm owner; thus, sampling was done by the farm owner himself/herself after clear instructions on the sampling procedure were given. Furthermore, the collection of all animal samples were approved by the ethical commission of the Animal Welfare Body Utrecht (protocol number: 10803–2025-01). The medical research ethics committee (MREC) of the University Medical Center Utrecht (UMCU) declared that our study was not subject to the Dutch WMO (Medical Research Involving Human Subjects Act), meaning that the research did not require review and approval by the MREC (protocol number: 23–284/dB).

### 2.2 Sample collection

After each farm visit, samples were taken to the laboratory at Utrecht University in a chilled environment (4 °C), processed in the laboratory and stored in the refrigerator within 24 h after sampling. See Supplementary material 2 for the exact field work protocol.

#### 2.2.1 Electrostatic dust collectors

Electrostatic dust collectors (EDCs) are passive sampling tools used to capture microorganisms in the air with an electrostatically charged cloth that attracts dust particles. The preparation of the EDCs was done by the design and protocol by Noss et al. [30] with some modifications that can be found in the Supplementary materials 3. On each farm, one EDC was placed in the goat stable above the goats, and one EDC was placed above the milking parlour. EDCs were hung at approximately 3 m high, measured 0.407m^2^ per sample and stayed in place for 5–7 days. In total, 50 EDCs were collected on 25 farms. In addition, one extra EDC was taken to each farm, but not taken out of its packaging as a field control.

At the laboratory, the protocol by Noss et al. [30] was followed with some modifications (Supplementary materials 4). Two aliquots of the sample were stored in 50% glycerol at −80 °C. One aliquot with only the sample, for DNA-isolation, was stored at −20 °C.

#### 2.2.2 Swabs (nose, hands, teat cups)

All samples were taken by the same researchers who followed the protocol briefly described below, except on two farms where access was not permitted and sampling was performed by the farm owners following instructions provided by the researchers. All the swab samples included in this study were taken using a Copan eSwab (480CESR, Copan, Brescia, Italy). In each farm, the noses of five random goats from the pen under/near the EDC in the stable were swabbed. The same swab was gently rubbed against the inside of the nasal passage of both nostrils. One swab was taken out of the package and held in the air for 10 s in the stable as a field control. In total, 145 goat nose swabs were collected on 29 farms. In addition, four completely unopened swabs were used as extra controls (closed control swabs). Furthermore, the hands of 1–4 farm workers/residents per farm were swabbed. Per participant, one swab was used for both hand palms and the inside of all fingers. If a participant wore gloves, this was reported, but gloves were not removed. Additionally, questions were asked to gain insight about the persons' glove use habits and their tasks, whereabouts, and time spent on the farm. In total, 80 hand swabs (4 with disposable gloves and 75 without gloves, and 1 was excluded because the same swab was used to sample both the gloved and ungloved hand of a single participant) were collected on 29 farms. In addition, two pairs of teat-cups from the milking parlour were sampled on each farm. In side-by-side parlours, one pair of teat cups was sampled from the front and one pair from the rear of the parlour, both on the right-hand side. In rotary parlours (carousels), one pair of teat cups was sampled from each of two opposing positions on the carousel. Swabbing was performed on both the internal (the rubber/silicone teat cup liner) and the external (handles) surfaces, using a single swab. In total, 55 goat teat cup swabs were collected on 28 dairy goat farms, one farm was not sampled due to human error.

In the laboratory, two aliquots of the sample were stored in 50% glycerol at −80 °C (see Supplementary materials 5 for the protocol). One aliquot, containing only the sample for DNA isolation, was stored at −20 °C.

### 2.3 *Staphylococcus aureus* culturing and testing

After the last farm visit in May 2025, all samples were retrieved from the freezers at Utrecht University and transported in a chilled environment (4 °C) to the Wageningen Bioveterinary Research laboratory (WBVR) for further processing.

The isolation and identification of *S. aureus* was carried out following the protocol by Exel et al. [9] with some modifications (Supplementary materials 6). *Staphylococcus aureus,* identified by a Bruker MALDI (Matrix-Assisted Laser Desorption/Ionization) Biotyper Sirius (Billerica, Massachusetts, US) using the database BDAL version 2023, was stored in DNA-free water at −20 °C, and *S. aureus* in 30% glycerol was stored at −80 °C.

### 2.4 Whole genome sequencing

In November 2025, the *S. aureus* samples were prepared at WBVR. First, the *S. aureus* colonies (saved in 150 μl DNA-free water) were taken out of the freezer to defrost before 100 μl TE buffer was added. Then, a total volume of 270 μl of the sample mixture was processed according to the MolGen procedures, first using the Prelysis Booster, followed by the PathExtract kit for cell lysis and DNA isolation. Finally, the DNA was eluted in 100 μl elution buffer. The quality and quantity of the isolated DNA were checked using the HS-DNA Quant-IT kit (ThermoFisher, Waltham, Massachusetts, US). For the sequencing library, the Illumina DNA Prep kit was used according to the manufacturer's protocol. Per isolate, 50 ng of DNA was used as input. After making the libraries, more quality checks were done with the HS-DNA Quant-IT kit (to measure the DNA quantity). Additionally, an Agilent TapeStation was used to examine the fragment profile and verify the size and spread of the libraries. Then the molarity of each *S. aureus* sample was determined to calculate the amount of each sample needed. Lastly, all the libraries were normalised, meaning the same amount of each sample was loaded on the Illumina NextSeq2000 (Illumina, Inc., San Diego, California, US). This way, each sample would have enough sequencing data for a reliable analysis. Six samples were excluded from WGS analysis due to incorrect storage.

### 2.5 Statistical analyses

All the data were entered into Microsoft Excel (Microsoft Corporation, Redmond, Washington, US), and the statistical analysis was conducted using IBM SPSS 27 (Statistical Package for the Social Sciences) (IBM, New York, US). See Supplementary material 7 for an overview of the variables in this study. Some variables were excluded. This can be read in Supplementary material 8. For the statistical analyses concerning *S. aureus,* a MALDI-TOF (Matrix-Assisted Laser Desorption/Ionization, Time-Of-Flight) identification) score of 2 or higher was considered reliable. All control swabs tested negative and were excluded from further analysis. The differences of *S. aureus* positive proportions between categorical variables (sample type) were examined using Pearson's Chi-squared test, and the significance was set to *p*-value = 0.05. Because of multiple pairwise comparisons between groups (5 different sample types), Bonferroni correction was applied (10 possible comparisons). For the sequencing data analysis, raw sequencing reads were quality filtered by removal of adapter sequences and trimming of bases with a Phred score below Q30. The resulting high-quality reads were assembled using SPAdes v4 [31]. Draft genomes were assessed for completeness and contamination using CheckM2 [32]. Genomes passing quality control were imported into Ridom SeqSphere for cgMLST-based comparative analysis [33]. Clusters were defined using a cgMLST allele distance threshold of ≤25, selected after inspection of the allele distance distribution to ensure inclusion of closely related isolates within farm-associated clusters.

## 3 Results

On average, 11.3 ± 1.1 (mean ± SD) samples were taken per farm. Each sample had on average 2.1 ± 1.0 distinct colony morphologies (mean ± SD). Two out of twenty-nine farms (6.9%) did not have any positive samples for *S. aureus*. In total, 88 (26.7%) of all samples on all farms were positive for *S. aureus*. Out of all the samples (329), 15% had no bacterial growth. A complete overview of the bacterial findings from the farm samples is presented in Supplementary material 9.

The percentage of *S. aureus* positive samples per farm ranged from 0.0% to 58.3% (mean 26.3%, SD 15.8%). See Supplementary material 10 for a detailed overview of the number of positive samples per farm.

Airborne dust samples in the milking parlour and deep litter stable had a higher proportion (>50%) of positive *S. aureus* samples compared to goat nose swabs, hand swabs and teat cup swabs (Fig. 1.; *p*-value of <0.001). One positive airborne dust sample was found in 48% of farms, 36% of farms had two positive dust samples, and 16% of farms had no positive dust samples.

**Fig. 1:**
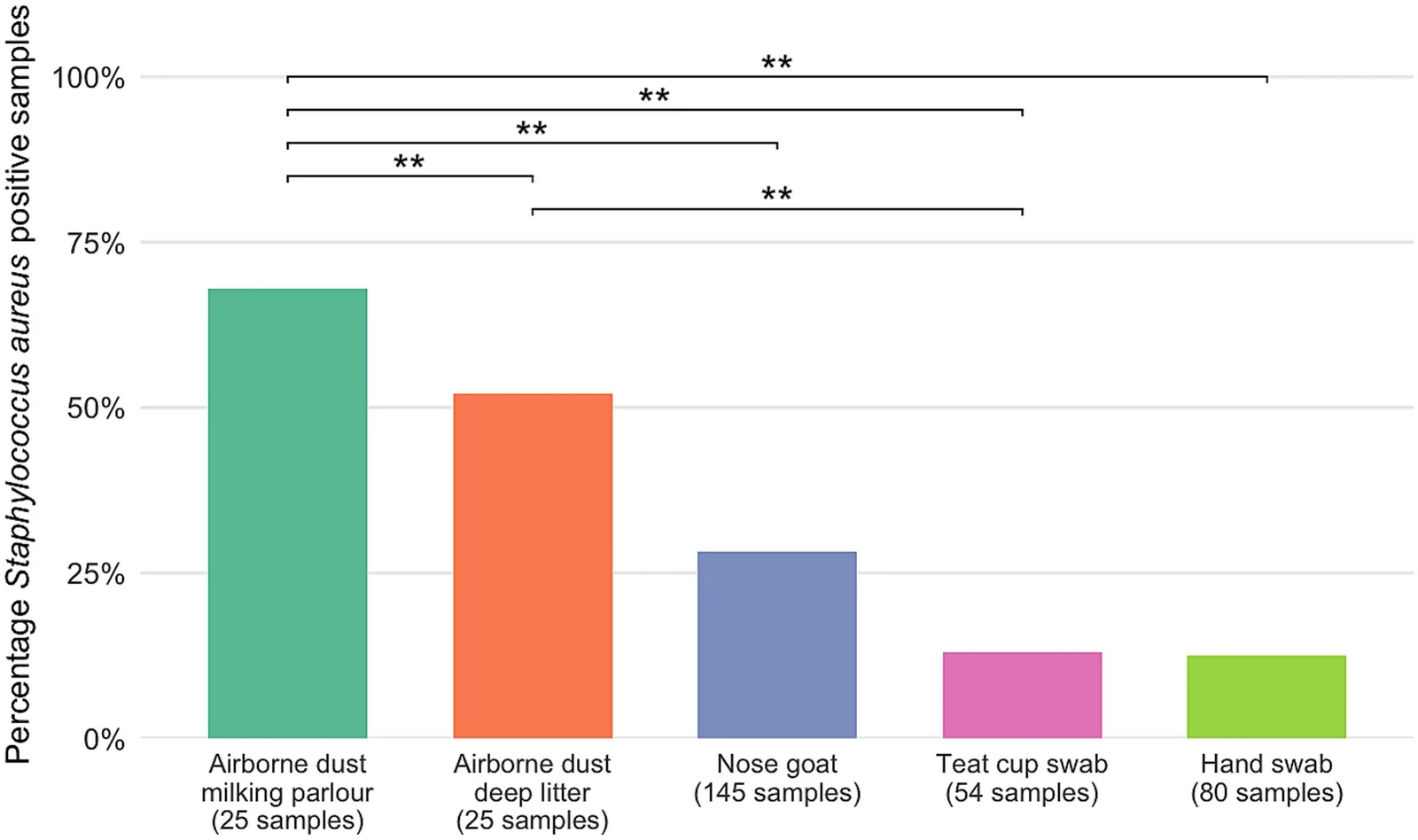
Proportion of *Staphylococcus aureus* positive and negative samples per sample type. Sample numbers shown below each bar indicate the total number of samples collected. Bar plots connected by a bracket with asterisks (**) indicate statistically significant differences between groups:*p* < 0.01. *P*-value was calculated, using Chi square tests with Bonferroni correction.

Sequencing identified five different sequence types (STs): ST133 was the most prevalent (76.8%), followed by ST398 (9.8%), ST7 (2.4%), ST45 (1.2%), and ST97 (1.2%), while 8.5% of the isolates could not be assigned a sequence type (NA) (Table 1). Interestingly, ST7 and ST45 were found in all samples except for the goat nose and airborne dust samples and ST398 was found in all samples except for the airborne dust samples.

**Table 1:** Overview of *Staphylococcus aureus* sequence types found in this study across sample types.

Sequence type *S. aureus*	Goat (nose)	Human (hands)	Environmental (teat cups)	Environmental (airborne dust)	Total samples n (%)
ST7		1	1		2 (2.4%)
ST45		1			1 (1.2%)
ST97	1				1 (1.2%)
ST133	32	4	5	22	63 (76.8%)
ST398	2	1	5		8 (9.8%)
NA⁎	5			2	7 (8.5%)
Total samples	40	7	11	24	82 (100%)

⁎ NA = sequence type not assigned.

Clustering analysis showed that isolates obtained from the goats' noses, farm workers' hands, teat cups, and EDCs were frequently grouped in the same clusters. No clear separation between these sample sources was observed, while isolates originating from the same farm more often clustered together (see Fig. 2.). A total of 14 farm-level clusters (C1-C14) were identified. Out of these clusters, 71% (10 clusters) contained only one farm, and 29% (4 clusters) included isolates from multiple farms. In addition, out of all samples, 73% (60 out of 82) lie within a cluster, while a substantial proportion of samples did not cluster with others (NC = 22). At the farm level, most farms contributed only a small number of clustered samples, and several farms had no clustered samples at all. For five samples (11.6% of the sequenced samples), multiple *S. aureus* isolates within the same sample were sequenced to evaluate within-sample variation and determine whether the *S. aureus* strains within a sample were genetically identical. While some samples contained identical strains, others showed genetic differences between isolates in the same sample (see Fig. 2.). In three instances, isolates within a sample were identical. In one of these, the sample did not cluster with other samples from the same farm. In another, the isolates clustered with some, but not all, samples from that farm. In the third, they clustered with all the samples from that farm. In two additional instances, isolates within a sample differed genetically, but still fell within the farm-specific cluster. For four farms in which all samples clustered within a single cluster, genetic distances between sample types were analysed. No consistent patterns were identified in distances between sample types within a cluster.

**Fig. 2:**
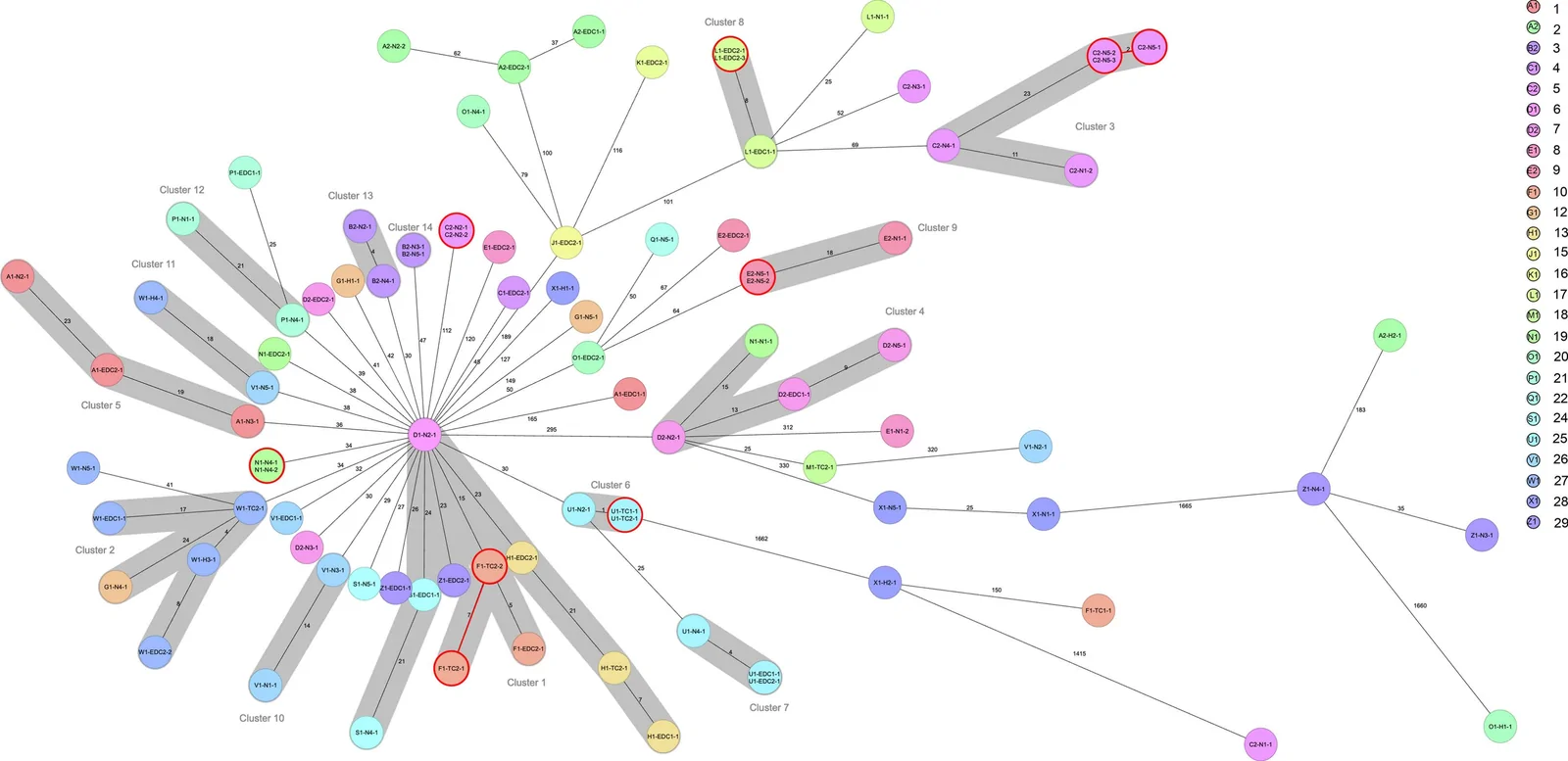
Clustering of *S. aureus* isolates based on cgMLST and coloured by farm. Sample IDs indicate the farm by a number from 1 to 29 and an additional number indicating the round. The type of sample is indicated with N (Goat nose), H (Human hands), TC (Teat cups of the milking parlour), EDC1 (Electrostatic dust collector in goat stable), or EDC2 (Electrostatic dust collector in the milking parlour stable). Branches are labelled with the cgMLST allele distances and clusters were defined using a threshold of ≤25. *Staphylococcus aureus* strains collected from one sample are circled and connected in red. (For interpretation of the references to colour in this figure legend, the reader is referred to the web version of this article.)

Participant characteristics for the 80 individuals providing hand swab samples are shown in Table 2. Most participants visited both the goat and the kids' stable, spent 2–4 h in the stable(s), had direct contact with goats/kids, and did not use gloves. Additionally, most participants were owners and were doing general tasks. Table 2 gives an overview of the descriptive test results of the proposed risk factors for *S. aureus*.

**Table 2:** Descriptive results of possible risk factors for *Staphylococcus aureus* carriage on hands with and without the use of gloves of farm workers at 29 Dutch dairy goat farms.

Variable⁎	Categories	Participants n (% of all participants within this variable)	*S. aureus* positive participants n (% positive of all participants within this category)
Stables visited	Both	40 (50.0%)	7 (17.5%)
	Dairy goats	26 (32.5%)	2 (7.7%)
	Kids	7 (8.8%)	1 (14.3%)
	None	7 (8.8%)	0 (0.0%)
Hours in stable(s)	0 h	7 (9.2%)	0 (0.0%)
	1–2 h	19 (25.0%)	2 (10.5%)
	2–4 h	28 (36.8%)	4 (14.3%)
	>4 h	22 (29.0%)	4 (18.2%)
Contact with goats/kids	Yes	61 (87.1%)	8 (13.1%)
	No	9 (12.9%)	0 (0.0%)
Glove use	Yes	27 (38.9%)	4 (17.4%)
	No	44 (61.1%)	4 (9.1%)
Role on farm	Owner	40 (51.3%)	7 (17.5%)
	Milker	15 (19.2%)	2 (13.3%)
	Employee	18 (23.1%)	1 (5.6%)
	Family member	5 (6.4%)	0 (0.0%)
Tasks	General tasks	50 (66.7%)	8 (16.0%)
	Milking	15 (20.0%)	2 (13.3%)
	Kids	6 (8.0%)	0 (0.0%)
	Non-stable tasks	4 (5.3%)	0 (0.0%)

⁎ For some variables, a few participants were excluded due to a lack of data.

## 4 Discussion

This research aimed to investigate the reservoirs and possible transmission routes of *S. aureus* on Dutch dairy goat farms using a One Health approach. *Staphylococcus aureus* is nearly universally present across Dutch dairy goat farms, highlighting its potential for transmission among goats, the farm environment, and humans. EDCs were used to study airborne dust contamination, as dust may act as a fomite to spread *S. aureus*. Human hands and teat cups were positive for *S. aureus* less frequently compared to airborne dust and goat nose swabs. Whole genome sequencing identified ST133 as the predominant sequence type, while ST398 was the next most frequently identified sequence type, with isolates clustering predominantly by farm across nose, hand, teat cup, and dust samples.

### 4.1 Prevalence of *Staphylococcus aureus*

Based on this study, *S. aureus* is frequently found on Dutch dairy goat farms, posing potential health risks for both goats and humans. Only 2 out of 29 farms tested negative for all samples. This is in line with previous research where 85% of goat farms tested positive for *S. aureus* in their bulk milk tank samples [34]. Across all sample types, the mean proportion of *S. aureus* positive samples was 27%. Since *S. aureus* was found on most farms and across many sample types, farm owners should assume *S. aureus* is likely present on their farm, even when clinical signs are not observed. This finding is supported by the fact that detection of subclinical mastitis infections in goats is challenging [35], [36]. *Staphylococcus aureus* may spread unnoticed among animals, thereby increasing risks for both animal health and human health. Possible options to improve hygiene to mitigate the risks on dairy goat farms have been discussed previously in detail by Tibebu et al. [6].

### 4.2 Environmental transmission

*S. aureus* was significantly more present in airborne dust samples compared to other sample types, with no differences in the proportion of positive samples between the deep litter stable and the milking parlour stable. Electrostatic dust collectors (EDCs) collected dust over a period of 5–7 days, whereas the swab samples represented only a single point in time. While there have been no published studies that have detected *S. aureus* with EDCs on goat farms, the results for this study are in line with previous research that was able to detect *S. aureus* with EDCs in other types of farms. For example, a study by Rittscher et al. [37] detected *MRSA* using quantitative PCR-based methods on 94% of EDCs on pig farms. Likely, the chance of finding *S. aureus* may differ for each sample type that was used in this study. In possible future research, a distinction between cleaned and uncleaned teat cups/hands is advised to be made, as this may help clarify their role as potential risk factors for the presence and transmission of *S. aureus*.

### 4.3 Whole genome sequencing

The observed clustering of isolates from goat noses, hands, teat cups, and airborne dust within the same clusters indicates that these sampling sites are closely connected within the farm environment. The absence of clear separation between these sample types suggests potential exchange of bacterial strains between humans, animals, and the farm environment. Clustering was largely farm-specific. This suggests that strains mainly circulate within farms rather than being widely shared between different farms. When looking for similarities across farms in the same cluster, the farms within each cluster did not share clear similarities in geographical location, herd size, or youngstock rearing strategy (on-farm versus off-farm), suggesting that these factors are unlikely to have contributed to their clustering. For future research, it would be valuable to explore whether farms within the same cluster share other underlying factors, such as the purchase of goats from another farm, shared feed suppliers, or common veterinarians. The identification of only one common sequence type (ST133) across multiple sample types supports the presence of a dominant strain within this study population. Previous studies have reported that *S. aureus* ST133 and ST398 have been found in small ruminants, with ST133 being the most prevalent [38], [39], [40]. These findings are consistent with the sequence types identified in this study. ST398, although traditionally considered livestock-associated, has been increasingly recognised as a cause of human infections [13], [41]. In contrast, while ST133 has also been detected in humans, their role in human disease remains poorly characterised [20]. Overall, these WGS findings provide evidence that genetically related *S. aureus* isolates are present across goat, human, and environmental samples within the same farm, suggesting that transmission predominantly occurs within, rather than between, farms.

### 4.4 Human exposure and risk factors

During this study, we asked participants what stables they had visited, how many hours they spent in the stable(s), if they had direct contact with goats/kids, if they used gloves, and what their role and tasks on the farm were. On this data, only descriptive statistics were done since the sample size is limited, and further analysis lies outside the scope of this research. Most participants visited both the goat and the kids' stable, spent 2–4 h in the(se) stable(s) and had direct contact with goats/kids. The high prevalence of *S. aureus* detected in airborne dust suggests that farm workers and residents may be repeatedly exposed to this bacterium during daily farm activities. Studies in pig and veal farming have demonstrated a correlation between airborne MRSA concentrations and MRSA carriage among farmers, suggesting that airborne exposure may contribute to bacterial colonisation [42]. Whether a similar relationship exists for *S. aureus* on dairy goat farms remains to be investigated. Most participants who entered the stable(s) did not wear gloves. Menzies [3] strongly advised using disposable gloves during milking. However, clear instructions and education on the proper use of gloves are essential, as incorrect practices (e.g. failing to clean or replace gloves after performing tasks that visibly contaminate them) and a false sense of safety may even facilitate the spread of bacteria instead of preventing it.

### 4.5 Strengths

One of the strengths of this study is the broad geographical representation. The focus is on goat farms from different parts of the Netherlands and gives a representative view of the sector, which increases relevance and applicability to the findings. Additionally, approximately the same number of samples were taken on each farm (the standard deviation is small), meaning each farm is equally represented in the dataset, reducing sampling bias. Another strength of this study is that data collection was not limited to interviews but also included direct on-farm observations and sampling. The researcher(s) visited all farms in person, allowing them to assess the environment, the goats, and staff practices firsthand. This strengthens the reliability of the data, as direct observation reduces the risk of bias from self-reported information by farm owners (for example, stated glove use) and provides a more accurate picture of actual practices. Another strength is that a culture method was used, which verifies both the bacteria's existence and vitality. This proves that the bacteria found were not only detectable but also alive and able to grow in the environment that was sampled. A major strength of this study is the use of WGS, which provides high-resolution genetic data allowing detailed comparison of *S. aureus isolates* across goat, human, and environmental samples, thereby strengthening conclusions regarding potential transmission pathways.

### 4.6 Limitations

One of the limitations of this study is the teat cup swabbing, which was performed on both the inside and outside surfaces of the teat cup. If future research on this is done, a differentiation between inside and outside of the teat cup needs to be made during sampling because these surfaces likely represent different routes of contamination. Second, for human samples, only the hands were swabbed, and no nose swabs were taken. This might have led to an underestimation of the prevalence of *S. aureus* in farm workers/residents since the nose is the most common reservoir according to Anderson et al. [8]. Another limitation is that, in two instances, samples were collected by the farm owners rather than the research team due to restricted access to the farms. Although the owners were provided with clear instructions on the sampling procedure, differences in sampling technique may have introduced variability in sample collection and should be considered when interpreting the results. Further, the bacteriological culture method, followed by MALDI-TOF, represents a relatively rough approach. The colonies on the plates were assessed visually, and the dominant growth of *Bacillus* across all samples seemed to mask the presence of *S. aureus* in some cases (see Supplementary material 11). *Staphylococcus aureus* may not have been detected despite being present, making the exact sensitivity of this culture-based approach uncertain and difficult to establish. Overall, for all statistical tests, farms were not analysed as separate groups. Instead, data were pooled across all 29 farms and grouped only by sample type. Because of this, farm-specific characteristics and dependence between observations of the same farms were not taken into account, even though considerable differences may exist between farms.

## 5 Conclusion

This study investigated the reservoirs and possible transmission routes of *Staphylococcus aureus* on Dutch dairy goat farms using a One Health approach. This study shows that *S. aureus* is probably present on nearly all Dutch dairy goat farms. *Staphylococcus aureus* was detected in dairy goats, humans, and the farm environment. It was most often found in airborne dust (on EDCs) in the deep litter stable and on EDCs in the milking parlour, and least often on hand swabs of farm workers/residents and teat cup swabs. Whole genome sequencing results suggest that *S. aureus* circulates within farms across goats, humans, and the environment, with specific strains dominating. These findings highlight that transmission of *S. aureus* is likely farm-specific, with dust and other environmental reservoirs contributing to its spread, emphasising the importance of control strategies targeting not only animals but also the farm environment. Measures focusing on hygiene practices, environmental management, and milking procedures may therefore be essential to limit spread. Exploring risk factors such as other bedding materials, which are less dusty, and ventilation strategies would provide further insight into transmission pathways.

## CRediT authorship contribution statement

**Mariam F.M. Adetunji:** Conceptualization, Data curation, Investigation, Methodology, Visualization, Writing – original draft. **Maxime A.M. Pals:** Conceptualization, Formal analysis, Investigation, Methodology, Resources, Supervision, Writing – review & editing. **Kacper Libera:** Conceptualization, Formal analysis, Methodology, Supervision, Writing – review & editing. **Andries A. Kampfraath:** Investigation, Methodology, Writing – review & editing. **Gerrit Koop:** Funding acquisition, Project administration, Supervision, Writing – review & editing. **Manouk Vrieling:** Project administration, Writing – review & editing. **Lidwien A.M. Smit:** Conceptualization, Writing – review & editing.

## Ethics statement

All animal samples were approved by the ethical commission of the Animal Welfare Body Utrecht (protocol number: 10803–2025-01). The medical research ethics committee (MREC) of the University Medical Center Utrecht (UMCU) declared that our study was not subject to the Dutch WMO (Medical Research Involving Human Subjects Act), meaning that the research did not require review and approval by the MREC (protocol number: 23–284/dB).

## Funding

This study was funded by a Public-Private Partnership (PPP) grant from the Dutch Ministry of Agriculture, Fisheries, Food Security and Nature through the TKI Agri&Food having the project title: LWV22218: “Improving udder health in dairy goats”; in Dutch “Verbetering uiergezondheid van melkgeiten” (BO-63-001-062). The Project is coordinated by Wageningen Bioveterinary Research, part of Wageningen University & Research, NL.

## Declaration of competing interest

The authors declare the following financial interests/personal relationships which may be considered as potential competing interests: Manouk Vrieling reports financial support was provided by Dutch Ministry of Agriculture, Fisheries, Food Security and Nature. If there are other authors, they declare that they have no known competing financial interests or personal relationships that could have appeared to influence the work reported in this paper.

## Data Availability

Data will be made available on reasonable request.
