# Supplementary material for: A cross-sectional study of Staphylococcus aureus reservoirs in dairy goats, their environment and farm workers
Source: One Health. 2026 Oct 17;23:101624. doi: 10.1016/j.onehlt.2026.101624 (PMC13734940; doi:10.1016/j.onehlt.2026.101624)
Supplement: Supplementary file 1 [file mmc1.docx]

**Supplementary material 1**

This figure (1) shows the locations of all participating farms (with random numbers attached to each farm).

**Supplementary Fig. 1.** Locations of participating farms.
