# Supplementary material for: A cross-sectional study of Staphylococcus aureus reservoirs in dairy goats, their environment and farm workers
Source: One Health. 2026 Oct 17;23:101624. doi: 10.1016/j.onehlt.2026.101624 (PMC13734940; doi:10.1016/j.onehlt.2026.101624)
Supplement: Supplementary file 2 [file mmc10.docx]

**Supplementary material 10**

This table (2) gives a detailed overview of the number of positive samples per farm. 

**Supplementary Table 2**

Proportion of positive *Staphylococcus aureus* (*S. aureus*) samples per farm.

| Farm ID | *S. aureus* present   n of positive samples on the farm   (expressed as % of all samples taken on the farm) | Total number of samples   (n, samples taken per farm) |
| --- | --- | --- |
| 1 | 5 (41.7%) | 12 |
| 2 | 4 (36.4%) | 11 |
| 3 | 4 (36.4%) | 11 |
| 4 | 1 (8.3%) | 12 |
| 5 | 7 (58.3%) | 12 |
| 6 | 1 (9.1%) | 11 |
| 7 | 5 (41.7%) | 12 |
| 8 | 2 (18.2%) | 11 |
| 9 | 3 (30.0%) | 10 |
| 10 | 5 (45.5%) | 11 |
| 11 | 0 (0.0%) | 9 |
| 12 | 3 (30.0%) | 10 |
| 13 | 3 (27.3%) | 11 |
| 14 | 0 (0.0%) | 10 |
| 15 | 1 (7.7%) | 13 |
| 16 | 1 (9.1%) | 11 |
| 17 | 3 (27.3%) | 11 |
| 18 | 1 (8.3%) | 12 |
| 19 | 3 (25.0%) | 12 |
| 20 | 3 (25.0%) | 12 |
| 21 | 3 (27.3%) | 11 |
| 22 | 1 (11.1%) | 9 |
| 23 | 1 (7.7%) | 13 |
| 24 | 3 (27.3%) | 11 |
| 25 | 6 (46.2%) | 13 |
| 26 | 5 (45.5%) | 11 |
| 27 | 6 (46.2%) | 13 |
| 28 | 4 (36.4%) | 11 |
| 29 | 4 (30.8%) | 13 |
| Total | 88 (26.7%) | 329 |
