# Supplementary material for: A cross-sectional study of Staphylococcus aureus reservoirs in dairy goats, their environment and farm workers
Source: One Health. 2026 Oct 17;23:101624. doi: 10.1016/j.onehlt.2026.101624 (PMC13734940; doi:10.1016/j.onehlt.2026.101624)
Supplement: Supplementary file 3 [file mmc11.docx]

**Supplementary material 11**

This figure (3) shows pictures of hemolysis patterns that suggest *S. aureus* growth hidden behind Bacillus growth.

**Supplementary Fig 3.** Hemolysis behind *Bacillus* growth (four examples)
