# Supplementary material for: A cross-sectional study of Staphylococcus aureus reservoirs in dairy goats, their environment and farm workers
Source: One Health. 2026 Oct 17;23:101624. doi: 10.1016/j.onehlt.2026.101624 (PMC13734940; doi:10.1016/j.onehlt.2026.101624)
Supplement: Supplementary file 4 [file mmc2.docx]

**Supplementary material 2**

This protocol describes the materials needed and procedures required for on-site sampling during farm visits and was considered to be as sterile as possible. The average sampling time per farm is 30 minutes.

Materials:

- Plastic gloves, boots, and overalls for each person
- Contract/consent form (to be signed by the farmer)
- Printed survey (accompanying the hand swabs)
- Pen and permanent marker
- 5x Nasal Copan eSwabs 480CE
- 4x Hand Copan eSwabs 480CE
- 1x Control Copan eSwab 480CE
- Tube rack
- 3x EDCs (Electrostatic Dust Collectors) from IRAS, cloths (area: 0.0209 m2) from Albert Heijn, heated at 200°C for 4 hours
- 2x Cardboard Box or plastic plank (big enough for EDC to fit in/on top of it)
- Rope, tyraps and scissors
- Cooler box with ice or cooling elements
- 70% Ethanol with cotton wool
- Trash bag
- Present/snack for the farm owner/interviewee/participant(s)

Sample types:

- Nasal swab from 5 different goats
- Farmworker/Farm resident hand swabs (maximum 4 people)
- Teat cup swabs from two pairs of teat cups (milking parlour)
- One EDC in the pen and one in the milking stable
- Controls (air swab and an unopened EDC)

Procedures:

Wear gloves, boots and an overal before entering the goat stable.

1. Nasal swab (goat)

Take the swab and gently rub it against the inside of one nasal passage (do not insert the swab deep into the nose). Repeat this for the other nasal passage using the same swab. Write down the number of the goats’ ear tag on the swab. Repeat this until five goats are sampled. Additionally, take one extra control swab, open it and hold it in the air inside the goat stable for 10 seconds. Write a ‘C’ on this swab.

Additionally, score the farm on multiple environmental factors in the stable: hot/clammy/study, ammonia smell, dust in air, dust on ground, dry eyes, blocked nose, sore throat. These factors need to be scored on a five-point scale: very much/extremely, much/clearly noticeable, moderate/average, little/hardly noticeable, not at all.

2. Hand swab (human)

Ensure the owner/manager of the farm has signed the contract/consent form. Swab both handpalms using the same swab, including all ten fingers. If the person is wearing gloves, make sure these are not removed. Complete the corresponding survey for each person swabbed. The questions are as follows:

- Is there currently a kidding period on the farm?
- What is your role and what are your tasks on the farm?
- How many hours did you spend in the stable today?
- Did you visit the milkgoats and/or the kids’ stable?
- Which stable did you visit last (dairy goat stable or kid stable)?
- How many hours did you touch the dairy goats today?
- How many hours did you touch the kids today?
- Did you wear gloves during your work?
- How many times did you wash your hands today?

Write down the location of sampling and whether the person was wearing gloves or not at the moment of sampling. Lastly, write down the code given to each person on both the swab and the survey (e.g. Farm A: H1, H2, H3, or H4).

3. Teat cup swab (milking parlour)

Do not touch the milking cups directly before taking the swab. Use the same swab to sample first the right teat cup, then the left teat cup of the pair. Brush the swab against the handles of the teat cup and the silicone/rubber inside liner of the teat cup. In side-by-side parlours, one pair of teat cups will be sampled from the front and one pair from the rear of the parlour, both on the right-hand side. In rotary parlours (carousels), one pair of teat cups will be sampled from each of two opposing positions on the carousel.

4. EDCs (pen and milking stable)

Hang one carton box/plastic plank at a suitable height around the goats in the pen and one in the milking stable (approximately 3 meters high). Hang them at a site where the goats cannot touch them and where there is minimal air disturbance. Carefully take the EDC out of its plastic packaging, fold it open and place it horizontally in the cardboard box (or on the plastic plank: use tape to hold the EDC in place on the plank). Do not touch the cloth at all times! Leave the EDCs in place for 5-7 days. Take one extra (control) EDC, but keep it sealed in its packaging. After the sampling period, take the EDCs without touching the cloth and place them in a labelled plastic bag.

5. Additional

Take photos of where in the pen and milking parlour the EDCs are placed, as well as of any other relevant details.

After sampling, put all the swabs in the tube rack and into the cooler box. Always wash your hands after coming out of the stable and clean all the materials taken into the stable with ethanol. Never use the same boots, overalls, gloves or materials at another farm before having cleaned everything.
