# Supplementary material for: A cross-sectional study of Staphylococcus aureus reservoirs in dairy goats, their environment and farm workers
Source: One Health. 2026 Oct 17;23:101624. doi: 10.1016/j.onehlt.2026.101624 (PMC13734940; doi:10.1016/j.onehlt.2026.101624)
Supplement: Supplementary file 5 [file mmc3.docx]

**Supplementary material 3**

The preparation of the EDCs used for this study followed the protocol by Noss et al. (2008) with some modifications. In this study, cloths (area: 0.0209 m2) were bought from a supermarket and sterilised in the laboratory at Utrecht University by heating them at 220°C in an oven for 4 hours before attaching them to an alcohol-cleaned polypropylene folder (40 x 30 cm). Per EDC, two cloths were used, thus a total of 0.407m² was measured per sample.
