# Supplementary material for: A cross-sectional study of Staphylococcus aureus reservoirs in dairy goats, their environment and farm workers
Source: One Health. 2026 Oct 17;23:101624. doi: 10.1016/j.onehlt.2026.101624 (PMC13734940; doi:10.1016/j.onehlt.2026.101624)
Supplement: Supplementary file 6 [file mmc4.docx]

**Supplementary material 4**

This protocol by Noss et al., (2008) with some modifications, describes the materials and equipment needed for saving the EDCs in the freezer. This process takes approximately 30 minutes for 2 EDCs. Gloves need to be worn during all steps of this protocol.

Materials:

- EDC(s) 🡪 from the pen, from the milking stable, or the control
- 0.85% NaCl + 0.05% Tween 80 in distilled water
- Stomacher bags 400 mL and clips
- Holder and rack for stomacher bags
- Stomacher machine
- 50 mL Falcon tubes and rack
- Micronics tubes with stickers + caps + Micronics block
- Beckman Coulter Allegra X-12R Centrifuge
- 50% glycerol
- Aluminum foil
- Tweezers
- 1000 µL pipette + tips
- 20 mL pipette
- Hirschmann Laborgenerate Pipetus (automatic pipette)
- -20°C freezer
- -80°C freezer

Procedure:

Clean the Table with ethanol and prepare all materials. Make sure all Mironics tubes have a sticker with the sample ID and are in the right order in the Mironics block.

Clean the tweezers with ethanol and prepare a labelled stomacher bag in the holder. Place a sheet of aluminium foil on the Table and open the EDC on top of it. Use sterile tweezers to take both the napkins from the EDC and place them into the stomacher bag. Add 20 mL of extraction buffer (0.85% sodium chloride (NaCl) + 0.05% Tween 80 in distilled water) using the automatic pipette. Hold the pipette above and not in the Stomacher bag so it can be reused. Close the bag with clips and place it in the rack. Repeat this step for the other EDCs.

Place the stomacher bags in the Stomacher for 10 minutes at 230 rpm. Place a maximum of two bags in the Stomacher at a time. Label the 50 mL Falcon tubes and transfer the liquid from the stomacher bags into a Falcon tube by squeezing out the contents. Centrifuge the tubes at 50g for 1 minute at 21 °C (the tubes in the centrifuge need to be mirrored). Take the tubes out of the centrifuge and place them in the tube rack without shaking them!

Make 3 aliquots per sample (2 with and 1 without 50% glycerol). Fill the Mironics tubes of the aliquots 1 and 2 with 311 µL of 50% glycerol with a 200 µL pipette. Pipette 660 µL with a 1000 µL pipette from the sample from the Falcon tube into the first aliquot with glycerol. Resuspend the glycerol with the sample by pipetting up and down 3 times. Repeat this step for the second aliquot with glycerol. Then pipette 1000 µL of the sample from the Falcon tube with a 1000 µL pipette into the third aliquot (the one without glycerol).

Store the Micronics tubes that contain both the sample and glycerol (aliquots 1 and 2) at -80°C. Store the Micronics tubes that contain only the sample (aliquot 3) at -20°C. Make sure to have a Microsoft Excel file with the layout of each Mironics block and the place of storage. Also, record all discrepancies (incorrect volumes, wrong aliquots, etc) here.
