# Supplementary material for: A cross-sectional study of Staphylococcus aureus reservoirs in dairy goats, their environment and farm workers
Source: One Health. 2026 Oct 17;23:101624. doi: 10.1016/j.onehlt.2026.101624 (PMC13734940; doi:10.1016/j.onehlt.2026.101624)
Supplement: Supplementary file 7 [file mmc5.docx]

**Supplementary material 5**

This protocol describes the materials needed for saving the swabs in the freezer. This process takes 30 minutes per batch (maximum of 12 swabs). Gloves need to be worn during all steps of this protocol.

Materials:

- Ethanol 70%
- Swabs with 1 mL of liquid and the sample in a tube rack
- Vortex
- Pipette of 1000 µL + tips
- Multichannel pipette (12-channel, nominal volume of 300 µL) + tips
- Micronics tubes with stickers + caps + Mironics block
- Glycerol 50%
- -20°C freezer
- -80°C freezer

Procedure:

Clean the Table with ethanol and prepare all materials. Make sure all Mironics tubes have a sticker with the sample ID and are in the right order in the Mironics block. Make 3 aliquots per sample (2 with and 1 without 50% glycerol). Fill the Mironics tubes of the first two aliquots with 141 µL of 50% glycerol with a 200 µL pipette.

Arrange the swabs in the tube rack in the same order as the Micronics tubes. Vortex all swabs for 10 seconds. Take one swab, briefly vortex it, uncrew the top and press the cotton swab against the inside of the tube in a circular motion to extract all the liquid from the swab. Discard the swab.

Pipette 920 µL of liquid with a 1000 µL pipette from the swab tube into the third aliquot tube (the one witout 50% glycerol) and place the corresponding swab tube back in the rack or discard it. Then pipette 300 µL (2x 150 µL) of the sample from the third aliquot into the tubes of the first aliquot containing glycerol using the multichannel pipette of 150 µL. Do not touch the glycerol with your pipette tips the first time (so you can go back into the third aliquot without glycerol the second time without changing the tips). After having added 300 µL, resuspend the sample well with the glycerol by pipetting up and down 3 times. Change the pipette tips and repeat for the second aliquot. Close the Mironic tubes with the caps.

Store the Micronics tubes that contain both the sample and glycerol (aliquots 1 and 2) at -80°C. Store the Micronics tubes that contain only the sample (aliquot 3) at -20°C. Make sure to have a Microsoft Excel file with the layout of each Mironics block and the place of storage. Also, record all discrepancies (incorrect volumes, wrong aliquots, etc) here.
