# Supplementary material for: A cross-sectional study of Staphylococcus aureus reservoirs in dairy goats, their environment and farm workers
Source: One Health. 2026 Oct 17;23:101624. doi: 10.1016/j.onehlt.2026.101624 (PMC13734940; doi:10.1016/j.onehlt.2026.101624)
Supplement: Supplementary file 8 [file mmc6.docx]

**Supplementary material 6**

This protocol was derived from the protocol by Exel et al. (2023), with minor modifications. This protocol describes the materials and equipment needed for the selective breeding of *Staphylococcus aureus*.

This process takes 5-6 days. Make sure all tubes and plates have the sample ID written on them (or have a sticker). Before starting, make sure the table is cleaned with 70% ethanol and clean gloves are worn. All the culture broths and media used in this study were prepared by a specialised unit of the WBVR lab (WBVR-Mediabereiding).

*Day 1*

Materials:

- Sample (in 50% glycerol)
- Pipette 1-200 µL + tips
- Muller Hinton Broth (BD 275730) 15% NaCl (J.T. Baker 0278)
- Vortex
- 37°C incubator

Defrost the samples by placing the Micronics blocks in water. Pipette the sample up and down 3x to homogenise before pipetting 150 µL of the sample in 5 mL Muller Hinton Broth 15% NaCl (in the laminar flow cabinet) with a 200 µL pipette. Vortex for 2 seconds and incubate for 22-24 hours at 37°C. Before incubating, make sure the caps are loose on the tubes.

*Day 2*

Materials:

- Pipette 1-1000 µL + tips
- Muller Hinton Broth (BD 275730) 15% NaCl (J.T. Baker 0278) with sample
- Muller Hinton Broth (BD 275730) 10% NaCl (J.T. Baker 0278)
- Vortex
- 37°C incubator

Tighten the caps on the tubes and vortex for 2 seconds. Then pipette 500 µL of the incubated sample into 5 mL Muller Hinton Broth 10% NaCl (in the laminar flow cabinet) with a 1000 µL pipette, vortex for 2 seconds, and incubate for 22-24 hours at 37°C. Before incubating, make sure the caps are loose on the tubes.

*Day 3*

Materials:

- Muller Hinton Broth (BD 275730) 10% NaCl (J.T. Baker 0278) with sample
- Soft disposable loop 10 µL
- Heart infusion agar BD 244400 with 5% sheep blood, Analytichem BTSG500 (HIS-plate)
- Vortex
- 37°C incubator

Tighten the caps on the tubes and vortex for 2 seconds. Then, plate 10 µL with a soft disposable loop (10 µL) of the incubated sample on half a Heart-infusion agar plate with 5% Sheep Blood (HIS-plate). Use a different soft disposable loop (10 µL) for the second and third strike on the agar plate and incubate for 22-24 hours at 37°C.

*Day 4*

Materials:

- HIS-plate with sample
- ‘Clean’ HIS-plate
- MALDI-TOF plate, wooden sticks, and paper templates (12x8)
- 80% TFA
- Bruker standard solvent from Sigma-Aldrich (50% ACN + 47,5% H₂O + 2,5% TFA)
- 70% Ethanol
- Demineralized water
- HCCA (α-Cyano-4-hydroxycinnamic acid), a portioned Matrix for MALDI-TOF-MS (from Bruker)
- Pipette 1µL + tips
- Pipette 1-1000 µL + tips
- Vortex
- Bruker MALDI Biotyper sirius
- 37°C incubator
- 7°C fridge

Count the number of morphologies on the plate. Select one colony from each morphology, draw a circle around each colony and add the associated number. Write down the number of different morphologies and which colonies have hemolysis or not per sample. Make sure the MALDI-TOF plate is clean. If not, clean the plate with ethanol using special tissues. Then clean with water while holding the plate directly under the jet using special tissues. Repeat the cleaning step with ethanol and the step with water one more time. Then Pipette 100 µL TFA (in the laminar flow cabinet) with a 200 µL pipette onto the MALDI-plate (do not touch with hands), spread it out/clean with special tissues. Then rinse the MALDI-plate with demineralised water and tap clean with special tissues. Put a colony from each morphology into the MALDI-TOF. Add 250 µL HCCA with a 1000 µL Pipette to the tube with Bruker standard solvent and vortex until the powder is fully dissolved (~1 minute). Pipette a drop of 1µL with a 10 µL Pipette on each dot of the MALDI-TOF plate and let the plate dry (by air or no longer than 30 min on the heating device). Put the MALDI-TOF plate in the MALDI-TOF. Check and save the results. Replate the *S. aureus* positive colonies on half HIS-plates and incubate for 22-24 hours at 37°C. Save the original agar plates with the positive colonies from day 3 at 4°C.

*Day 5*

Materials:

- Eppendorf tube 1000 µL
- Micronics tubes + caps
- UltraPure distilled water Dnase/Rnase Free
- Pepton glycerol medium (Bacto Peptone BD 211677) + 30% glycerol (Merck 1.04094)
- Disposable loops 1 µL
- MWE MW102 Dryswabs
- -20°C freezer
- -80°C freezer
- 7°C fridge
- (37°C incubator)

Take the HIS-plates with *S. aureus* from day 4 out of the incubator. Fill one Eppendorf tube with 1000 µL Pepton glycerol medium + 30% glycerol with a 1000 µL Pipette (not in the laminar flow cabinet) and one Micronics tube with 150 µL DNA-free water with a 200 µL Pipette (in the laminar flow cabinet). Make sure the HIS-plate with *S. aureus* is a pure culture and no other bacteria are present. If not, replate this strain and incubate for 22-24 hours at 37°C (use the weekend incubator). If it is a pure culture, take a disposable loop (1 µL) and scrape one line of the bacteria off the plate and into the Micronics tube until the liquid turns cloudy. Then vortex for 5 seconds. Use a MWE MW102 Dryswab to scrape as much of the bacteria left on the HIS-plate off and put it in the Eppendorf tube until the liquid turns cloudy. Then vortex for 5 seconds. *Staphylococcus aureus* in DNA-free water needs to be stored at -20°C. *Staphylococcus aureus* in glycerol needs to be stored at -80°C. Save the strains under Ketzoo-number: SA.2025.... on the WUR-drive. Make sure each sample has its own Ketzoo number on the sticker of each tube. Ensure to have a Microsoft Excel file with the layout of each block, with the tubes and the place of storage of this block. Also, record all discrepancies (incorrect volumes, wrong aliquots, etc) here.

**weekend, 2 days off**

*Day 6 (Only if not all plates with S. aureus were of a pure culture on day 5!)*

Take the HIS-plates with *S. aureus* out of the incubator and repeat the steps from day.
