# Supplementary material for: A cross-sectional study of Staphylococcus aureus reservoirs in dairy goats, their environment and farm workers
Source: One Health. 2026 Oct 17;23:101624. doi: 10.1016/j.onehlt.2026.101624 (PMC13734940; doi:10.1016/j.onehlt.2026.101624)
Supplement: Supplementary file 9 [file mmc7.docx]

**Supplementary material 7**

The data used in this study includes several variables, each consisting of multiple categories. A clear overview of these variables is shown in Table 1. For some variables/categories, a slightly more detailed description is needed. This is provided below.

**Supplementary Table 1**

Overview of variables and categories used in the study.

| **Variable** | **Categories** | **Notes** |
| --- | --- | --- |
| Sample types | *EDC deep litter; *EDC milking parlour; Hand swab; Nose goat swab; Teat cup swab | - |
|  |  |  |
| Stables visited | Dairy goats; Kids; Both; None | Participants reported stables visited since start of day until sampling |
|  |  |  |
|  |  |  |
| Hours in stable | 0 h; 1–2 h; 2–4 h; >4 h | Self-reported time spent since start of day until sampling |
|  |  |  |
| Contact | Yes; No | Self-reported contact since start of day until sampling. Initially 4 categories (Dairy goats; Kids; Both; None), reduced to 2 (reason in Discussion) |
|  |  |  |
| Gloves | Yes; No | Partial glove use (n=15) classified as 'Yes' |
|  |  |  |
| Sample type | Skin; Gloves | Indicates swabbing location (skin versus gloves) |
|  |  |  |
|  |  |  |
|  |  |  |
| Role on farm | Owner (incl. Co-owner/Manager); Milker; Employee (incl. Intern); Family member | Family members classified based on tasks performed |
|  |  |  |
|  |  |  |
|  |  |  |
|  |  |  |
| Sampling location | Stable (incl. changing room); Outside; Canteen (incl. kitchen/office) | - |
|  |  |  |

*EDC = Electrostatic dust collector.

If a family member’s task was milking, they were assigned to the farm role category of ‘Milker’. If a family member did any tasks on the farm other than only milking, they were assigned to the category ‘Employee’. Family members without any farm tasks were categorised as ‘Family member’.
