# Supplementary material for: A cross-sectional study of Staphylococcus aureus reservoirs in dairy goats, their environment and farm workers
Source: One Health. 2026 Oct 17;23:101624. doi: 10.1016/j.onehlt.2026.101624 (PMC13734940; doi:10.1016/j.onehlt.2026.101624)
Supplement: Supplementary file 10 [file mmc8.docx]

**Supplementary material 8**

Data on handwashing habits and whether the farm was in a kidding period at the moment of sampling were collected but excluded due to limitations in data collection. Participants were also asked which stable they had last visited (the kids’ stable or the goat stable), which has been excluded from the study since it was not deemed to be as important. Furthermore, the variable ‘Sampling location’ of participants was not included in the statistical analyses of this study.
