# Supplementary material for: A cross-sectional study of Staphylococcus aureus reservoirs in dairy goats, their environment and farm workers
Source: One Health. 2026 Oct 17;23:101624. doi: 10.1016/j.onehlt.2026.101624 (PMC13734940; doi:10.1016/j.onehlt.2026.101624)
Supplement: Supplementary file 11 [file mmc9.docx]

**Supplementary material 9**

This figure (2) presents all the bacterial findings of this study.

**Supplementary Fig. 2.** Bacterial findings in this study. Some samples contained multiple bacteria; therefore, percentages do not sum to 100%.

Colonies thought to be *Bacillus* after visual examination were not intentionally put on the MALDI-TOF, meaning there are no MALDI-TOF results to support the identification of *Bacillus* or to give a specific *Bacillus* type in the majority of the cases. In some cases, the MALDI-TOF (not intentionally) identified Bacillaceae taxa (*Bacillus licheniformis*, a *Virgilbacillus halodenitrificans*, a *Virgilbacillus olivae*, and an Oceanobacillus onocorhynchi), which were all categorised into the category ‘*Bacillus’*. Moreover, 9% of samples contained other types of bacteria than the ones already named, these being: *Staphylococcus cohnii*, *Staphylococcus microti*, *Staphylococcus pettenkoferi*, *Staphylococcus succinus*, *Staphylococcus warneri*, *Streptococcus mitis_oralis*, *Steptococcus sanguinis*, *Brevibacterium ravenspurgense*, *Neisseria_sicca group*, *Staphylococcus arlettae*, *Mammaliicoccus fleurette*, and *Rothia aeria*. Only 5% of samples had no bacterial growth at all. Furthermore, the MALDI-TOF could not identify one or more morphologies in 16% of the samples and gave ‘No peaks’ for a morphology in two of the samples.
